# The effect of surgery on the survival status of patients with locally advanced cervical cancer after radiotherapy/chemoradiotherapy: a meta-analysis

**DOI:** 10.1186/s12885-018-4232-x

**Published:** 2018-03-20

**Authors:** Dan Shi, Zhenzhen Liang, Cheng Zhang, Huaiyu Zhang, Xiaodong Liu

**Affiliations:** 1 Department of Radiation Oncology, China-Japan Union Hospital of Jilin University, Changchun, China; 2 Epidemiology and Statistics, School of Public Health, Jilin University, Changchun, Jilin China; 3 Department of Cardiology department, China-Japan Union Hospital of Jilin University, Changchun, China; 4 Department of Gastrointestinal Colorectal and anal surgery, China-Japan Union Hospital of Jilin University, Changchun, China; 5 School of Public Health and Management, Wenzhou Medical University, Wenzhou, China

**Keywords:** Cervical cancer, Locally advanced disease, Radiotherapy, Surgery

## Abstract

**Background:**

To determine the effect of surgery on the survival status of patients with locally advanced cervical cancer after radiotherapy/chemoradiotherapy.

**Methods:**

PubMed, Web of Science, ProQuest and Medline were searched using the key words “cervical cancer”, “locally advanced disease”, “radiotherapy” and “surgery or hysterectomy”. Eight articles were selected and analysed using the STATA 12.0 software package. The log hazard ratio (HR) and its standard error for overall survival were calculated to assess the effect of surgery on patients with locally advanced cervical cancer after radiotherapy/chemoradiotherapy.

**Results:**

In total, 2176 patients with locally advanced cervical cancer were identified. The pooled HR for overall survival was 1.13 (95% confidence interval (CI) 0.906–1.409), and there were no differences among the eight manuscripts (z = 1.08, *p* = 0.278). In the subgroup analysis, the pooled HR for overall survival was 1.169 (95% CI 0.924–1.480), and no differences among patients with stage IB-IIB disease were found in six articles (z = 1.30, *p* = 0.193). There was no publication bias regarding overall survival or stage IB-IIB disease.

**Conclusions:**

This meta-analysis suggested that surgery had no effect on overall survival after radiotherapy/chemoradiotherapy; therefore, it is not recommended for patients with locally advanced cervical cancer.

## Background

Cervical cancer is the second-leading cause of cancer death in females, especially in less developed countries [1]. In 2012, there were approximately 527,600 new patients diagnosed with cervical cancer and 265,700 deaths caused by cervical cancer worldwide [2]. Disease stage is determined according to the 2009 FIGO (International Federation of Gynecology and Obstetrics) classification. Hysterectomy is performed for early stage disease, and chemotherapy and/or radiotherapy are administered based on the pathology results after surgery. For locally advanced cervical cancer, chemoradiotherapy is recommended as the standard treatment strategy because of its effectiveness at improving local control (LC) and reducing distant metastasis [3].

According to the 2017 National Comprehensive Cancer Network (NCCN) guidelines (part CERV-B two OF seven, "PRINCIPLES OF EVALUATION AND SURGICAL STAGING") [4], patients with stage IB2, IIA2 or IIB disease can first receive adjuvant chemotherapy and then undergo hysterectomy in some countries and regions. Radiotherapy or chemoradiotherapy can be administered depending on the pathology results after hysterectomy. The tumour volume could decrease after chemotherapy. In patients with stage IIB disease, parametrial invasion can be eliminated with chemotherapy. Patient can undergo surgery if the tumour shrinks and/or the parametrium becomes negative after adjuvant chemotherapy. As chemotherapy can reduce the tumour burden, we were curious what role radiotherapy or chemoradiotherapy plays in cervical cancer treatment. In addition, according to NCCN 2017, patients with stage IB2 and IIA2 disease can receive external pelvic radiotherapy combined with brachytherapy, followed by hysterectomy for those patients whose extent of disease or uterine anatomy precludes adequate coverage by brachytherapy as category III. We considered whether surgery is a better option for cervical cancer patients after radiotherapy or chemoradiotherapy, especially those with stage IB2, IIA2 and IIB disease. There are some articles on the application of surgery/hysterectomy after radiotherapy/chemoradiotherapy for locally advanced cervical cancer at stages IB2, IIA2, IIB, IIIA, IIIB, and IVA. This comprehensive meta-analysis was conducted to analyse the effect of surgery on survival status.

## Methods

This meta-analysis was performed according to the Preferred Reporting Items for Systematic Reviews and Meta-Analyses (PRISMA) guidelines [5].

### Search strategy

We identified studies in PubMed, Web of Science, ProQuest and Medline based on combinations of the following keywords: “cervical cancer” (“cervical tumor”, “cervical neoplasm”, or “cervical carcinoma”), “locally advanced disease”, “radiotherapy” (or “radiation therapy”), and “surgery or hysterectomy”. The most recent article was updated on August 9th, 2017. We also manually searched the references of related articles in this analysis.

### Inclusion/exclusion criteria

Studies were considered eligible if they met the following inclusion criteria: (1) The studies involved patients with locally advanced, non-metastatic cervical cancer at different stages (IB2, IIA2, IIB, IIIA, IIIB) and of different histological types. (2) The patients underwent radical/ extrafascial/ exploratory hysterectomy after radiotherapy/chemoradiotherapy, including external radiotherapy with/without brachytherapy. (3) For overall survival, the hazard ratio (HR) was compared, and the outcomes evaluations were well described. Kaplan-Meier curves or necessary data for calculating the log hazard ratio (logHR) and its standard error (SElogHR) were provided. (4) The median follow-up time was longer than six months. (5) The articles were written as full papers in English.

Studies were excluded for the following reasons: (1) The publications were review articles, letters, case reports, expert opinions, or meeting records. (2) Non-human research was performed. (3) Patients had recurrent or metastatic disease. (3) Key information for calculating logHR and SElogHR was missing. (4) The publications were not written in English.

### Data extraction

To avoid the repeated inclusion of the same data, the largest study with the longest follow-up time was included if there were several published studies involving the same patients at the same research centre. We included one study if different patients were included in two studies at the same research centre. Similarly, when there were multiple sets of data in one study, such as subsets of patients with different stage disease, we listed all data in separate sets. For data extraction, eligible articles were reviewed independently by two investigators. Discrepancies were resolved by discussion between the reviewers prior to data extraction. In cases of differing opinions, a third reviewer was consulted to reach consensus.

Multivariate and univariate Cox hazard regression analyses from publications were included in our analysis; if these results were not available, we extracted data from Kaplan–Meier curves of survival outcomes with log-rank *p* values or from survival plots and estimated logHR and SElogHR values provided by the authors.

Additional data were carefully extracted from all the eligible publications using a standardized data collection form, including first author, publication year, patient resource, histology, tumour stage, follow-up period, chemotherapy regimen, surgery type and other important clinical characteristics.

### Statistical methods

The logHR and SElogHR were used to analyse survival. We calculated the available statistics from published data using the methods developed by Parmar et al. [6], Tierney et al. [7], and Williamson et al. [8]. The calculable data included (i) multivariate and univariate Cox hazard regression analysis data with log-rank *p*-values and (ii) Kaplan–Meier survival curves with log-rank *p*-values. These data included the HR and 95% confidence interval (CI) directly cited in the related articles. Data from Kaplan–Meier survival curves with log-rank *p*-values were analysed using software designed by Matthew Sydes and Jayne Tierney [7] of the Medical Research Council Clinical Trials Unit, London, UK. Forest plots were used to estimate the role of surgery in patients with locally advanced cervical cancer.

Heterogeneity was defined as *p ≤* 0.10 or I^2^ > 50%. When homogeneity was present (*p* > 0.10, I^2^ ≤ 50%) [9], a fixed effect model was used for secondary analysis. An observed HR > 1 indicated a worse outcome for the surgery group. Publication bias is a major concern for all meta-analyses. Funnel plots were generated to assess potential publication bias, and *p* > 0.05 indicated no potential publication bias [10]. All statistical analyses were conducted using the STATA 12.0 software package.

## Results

### Characteristics of the identified studies

**Fig. 1:**
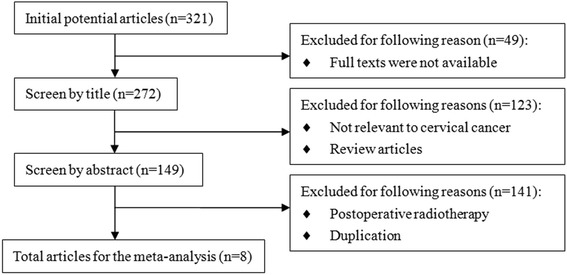
Flow chart of study identification. A search of the PubMed, Web of Science, ProQuest and Medline databases yielded 321papers. After selection according to the inclusion criteria, eight eligible studies were included in the meta-analysis

According to our previously defined criteria, the initial electronic online search of the PubMed, Web of Science, ProQuest and Medline databases retrieved 321 papers. After review according to the inclusion criteria, eight eligible studies were finally identified (Fig. 1).

The eight manuscripts included a total of 2176 patients with locally advanced cervical cancer. According to Darus 2008, the patients received surgery after external beam radiotherapy without brachytherapy. In Perez 1995, patients with stage IB, IIA, and IIB were included, which did not follow the 2009 FIGO guidelines. In this article, “bulky disease” was defined as a tumour diameter greater than five centimetres, in contrast to four centimetres in the 2009 FIGO guidelines. The overall survival data were available for patients in each stage. There were 1201 total cases in this study; 79 patients who received postoperative radiotherapy were excluded according to the defined criteria, and the remaining 1122 patients were included in the meta-analysis. Concurrent chemotherapy was not administered in this study (Table 1).

**Table 1:** Main characteristics of the studies included in the meta-analysis

Study	Country	Histology	Follow-up (months)	Stage	Chemotherapy regimen	CRT alone	CRT + surgery	Surgery type
Mazeron (2016) [17]	France	Squamous/Adenocarcinoma/Adenosquamous	39.3–75.5	IB1	Cisplatin /Carboplatin	157	54	Radical hysterectomy
				IB2				
				IIA2				
				IIB				
Fanfani (2016) [18]	Italy	Squamous/Adenocarcinoma	CRT: 14–127 CRT+ Surgery: 13–210	IIIA	Cisplatin	77	73	Radical hysterectomy
				IIIB				
Cetina (2013) [16]	Mexico	Squamous/Adenocarcinoma/Adenosquamous	3–80	IB2	Cisplatin and Gemcitabine	100	111	Radical hysterectomy
				IIA2				
				IIB				
Leguevaque (2011) [12]	France	Squamous/Adenocarcinoma	CRT: 5–64 CRT+ Surgery: 5–134	IB1	Cisplatin	44	67	Piver II extended/extrafascial hysterectomy
				IB2				
				IIA				
				IIA2				
				IIB				
				IIIA				
				IIIB				
				IVA				
Keys (2003) [13]	U.S.A.	Squamous/Adenocarcinoma/Adenosquamous	3.6–193.2	IB2	Cisplatin	124	132	Extrafascial hysterectomy
Morice (2012) [15]	France	Squamous/Adenocarcinoma	4.8–69.6	IB2	Cisplatin	30	31	Laparoscopic/radical hysterectomy
				IIA				
				IIB				
Darus (2008) [19]	U.S.A.	Squamous/Adenocarcinoma/Adenosquamous	1.5–138	IB2	Cisplatin, 5FU of capecitabine	30	24	Extrafascial hysterectomy
Perez (1995) [14]	U.S.A.	Epidermoid/Adenocarcinoma/Adenosquamous	36–276	IB	no	895	227	Exploratory laparotomy
				IIA				
				IIB				

*Chemo* chemotherapy

*CRT* chemoradiotherapy

### Assessment of methodological quality

We assessed the methodological quality of the included studies based on the Newcastle-Ottawa Scale (NOS; star system; range, zero to nine stars) [11] for the quality of cohort studies in a meta-analysis. In the current study, we considered a study awarded seven or more stars as a high-quality study because standard validated criteria for important end points have not been established. The ranking of each study is shown in Table 2. The NOS scores in the column titled “Comparability of Cohorts on the Basis of the Design or Analysis” indicate that some of the studies provided details regarding their design. In the studies by Leguevaque [12], Keys [13], and Perez [14], basic patient information was provided, but *p*-values were missing; hence, a star could not be awarded. According to the final results, the manuscripts in this meta-analysis were considered high-quality studies.

**Table 2:** Quality ratings based on the Newcastle-Ottawa quality assessment scale of the eight included studies

	Selection (score)				Comparability (score)	Outcome (score)			Total Score
	Representativeness of the Exposed Cohort	Selection of the Non-Exposed Cohort	Ascertainment of Exposure	Demonstration that Outcome of Interest was Not Present at Start of Study	Comparability of Cohorts on the Basis of the Design or Analysis	Assessment of Outcome	Was Follow-Up Long Enough for Outcomes to Occur	Adequacy of Follow-Up of Cohorts	
Mazeron (2016) [17]	1	1	1	1	2	1	1	1	9
Fanfani (2016) [18]	1	1	1	1	2	1	1	1	9
Cetina (2013) [16]	1	1	1	1	2	1	1	1	9
Leguevaque (2011) [12]	1	1	1	1	0	1	1	1	7
Keys (2003) [13]	1	1	1	1	0	1	1	1	7
Morice (2012) [15]	1	1	1	1	2	1	1	1	9
Darus (2008) [19]	1	1	1	1	2	1	1	1	9
Perez (1995) [14]	1	1	1	1	0	1	1	1	7

### Overall survival (OS) after radiotherapy/chemoradiotherapy with or without subsequent surgery

#### Overall survival analysis

**Fig. 2:**
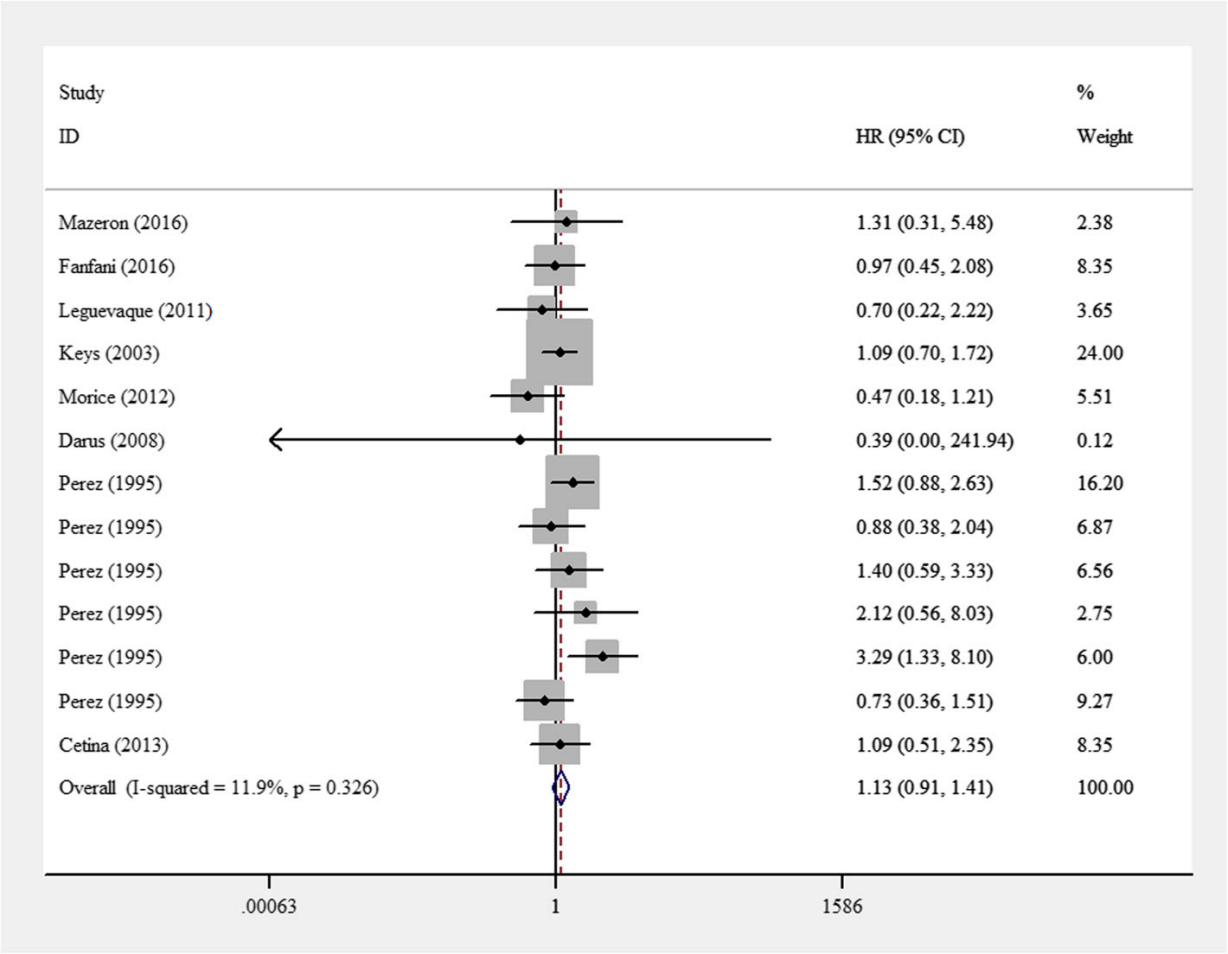
Results of the meta-analysis of overall survival of locally advanced cervical cancer patients. Hazard ratios for OS were available in eight studies with a total of 2176 patients. The pooled HR for OS was 1.13 (95% CI 0.906–1.409). The heterogeneity among studies was not high (*I*^*2*^ = 11.9%, *p* = 0.326). The pooled estimated HR for all studies showed no significant difference (z = 1.08, *p* = 0.278)

HRs for OS were available in eight studies for a total of 2176 patients. The pooled HR for OS was 1.13 (95% CI 0.906–1.409). The heterogeneity among studies was not high (*I*^*2*^ = 11.9%, *p* = 0.326). The pooled estimated HR for all studies showed no significant difference (z = 1.08, *p* = 0.278) (Fig. 2).

#### Subgroup overall survival analysis

**Fig. 3:**
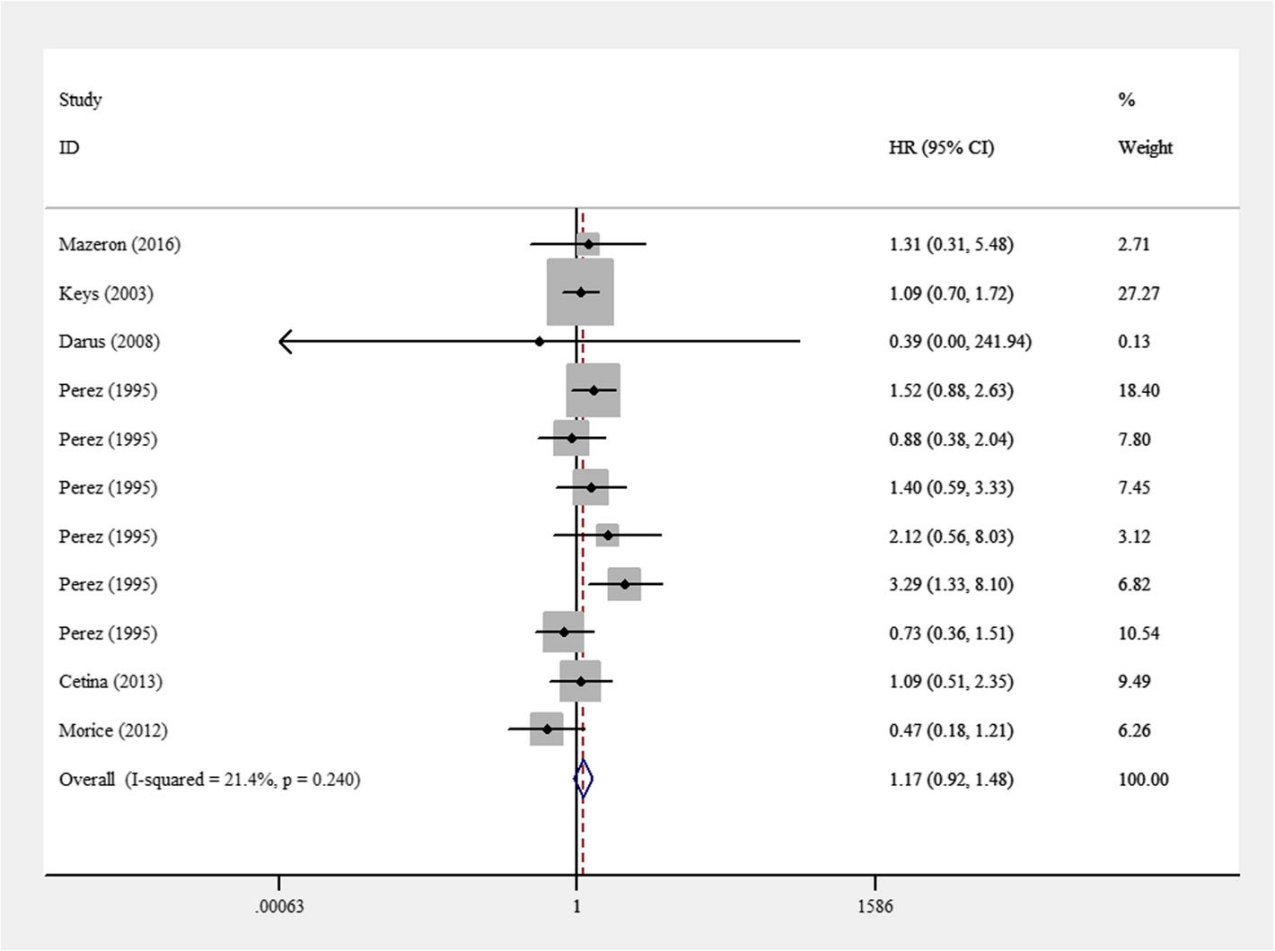
Meta-analysis results for the overall survival of subgroups of patients with locally advanced cervical cancer. Subgroup analysis was performed based on stage IB-IIB disease. Hazard ratios for OS were available in six studies with total of 1915 patients. The pooled HR for OS was 1.169 (95% CI 0.924–1.480). The heterogeneity among studies was not high (*I*^*2*^ = 21.4%, *p* = 0.240). The pooled estimated HR for all studies showed no significant difference (z = 1.30, *p* = 0.193)

A subgroup analysis was performed for patients with stage IB-IIB disease. HRs for OS were available in six studies for a total of 1915 patients. The pooled HR for OS was 1.169 (95% CI 0.924–1.480). The heterogeneity among studies was not high (*I*^*2*^ = 21.4%, *p* = 0.240). The pooled estimated HR for all studies showed no significant difference (z = 1.30, *p* = 0.193) (Fig. 3).

### Publication bias analysis

**Fig. 4:**
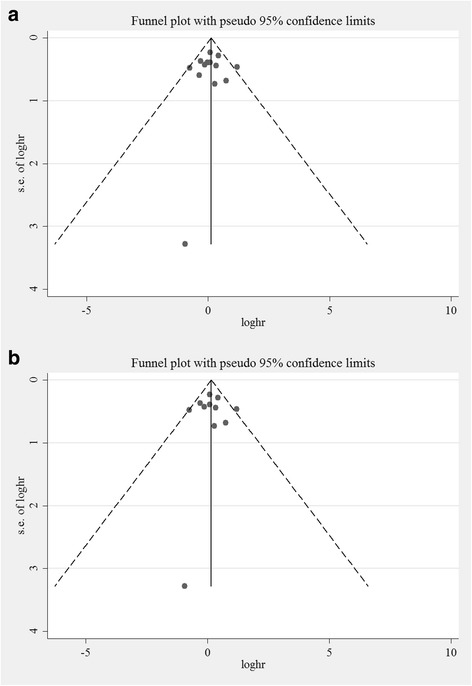
**a** and **b** Funnel plots for publication bias. Funnel plots were generated to assess publication bias. These plots showed obvious symmetry and no publication bias in the studies

Funnel plots were generated to assess the publication bias of the studies. These plots showed obvious symmetry and no publication bias (Fig. 4a and b).

## Discussion

According to the NCCN recommendations for early cervical cancer, hysterectomy is indicated with dissection of the cervix, corpus, parts of the vagina and ligaments, and pelvic lymph nodes. In this meta-analysis, radical, extrafascial, or laparotomy hysterectomy with lymphadenectomy was completed within six to 12 weeks after chemoradiotherapy/radiotherapy in eight studies. We conducted this meta-analysis to evaluate the role of surgery in patients with locally advanced cervical cancer after chemoradiotherapy/radiotherapy. Survival status and side effects related to surgery were examined.

### Clinical features

Radiotherapy/chemoradiotherapy followed by surgery could have different effects on survival status compared with chemoradiotherapy alone in patients with different stage disease in the eight manuscripts. However, surgery could have no effect on OS after radiotherapy/chemoradiotherapy.

According to Perez [14], the five-year cause-specific survival of patients with bulky IB2 (>five centimetres), IIA (>five centimetres) or IIB disease was 61%, 63%, and 69%, respectively, after radiotherapy and 60%, 72%, and 65%, respectively, after radiotherapy followed by surgery (*p* > 0.5). The ten-year OS of patients with bulky IB2 (>five centimetres), IIA (>five centimetres) or IIB disease was 61%, 68%, and 69% after radiotherapy and 44%, 72%, and 65% after radiotherapy followed by surgery (*p* > 0.5). Recurrence and metastasis rates were reported for the subgroups as follows: stage IB: 10% and 14%; stage IIA: 17% and 20%; and stage IIB: 23% and 29%. Chemotherapy was not utilized in this study. A tumour diameter greater than five centimetres was considered bulky disease; this definition differs from that stated in the 2009 FIGO guidelines (>four centimetres). For those patients with a tumour diameter more than four centimetres but less than five centimetres, the survival status was not included. These results are worth exploring. According to Keys [13], the five-year disease-free survival (DFS) and local recurrence (LR) rates were 62% and 53% in the surgery group and 14% and 27% in the chemoradiotherapy group for patients with stage IB2 disease (*p* > 0.5). Surgery could reduce the LR rate, especially among those patients with four-, five- and six-centimeter tumours. In the study by Leguevaque [12], the two-year DFS and recurrence rates were 66% and 49.7% in the surgery group and 22.4% and 36.4% in the chemoradiotherapy group (*p* < 0.05). The pelvic region was the main site of recurrence, occurring in 46.7% of the surgery group at 14 months and 56.2% of the chemoradiotherapy group at 11 months. The death rate caused by cervical cancer was 16.4% in the surgery group and 20.4% in the chemoradiotherapy group (*p* < 0.05), of which 45.4% and 77.8% of these cases were caused by pelvic recurrence (PR). As mentioned in this article, surgery was conducted in only patients who had a complete response (CR) or a residual tumour less than 50% of the initial size after chemoradiotherapy. Surgery did not improve OS but did increase DFS. In the study by Morice [15], the three-year OS and event-free survival rates of 86% and 97%, respectively, and 72% and 89%, respectively, were not significantly different according to surgery group among CR patients after chemoradiotherapy. Progression-free survival (PFS) and OS were similar between the two groups (74.8% and 71.7% for chemoradiotherapy alone; 76.3% and 74.5% for surgery after chemoradiotherapy) in the study by Centina [16]. According to Mazeron [17], the five-year DFS was 75.6% and 77.4% in the two treatment groups (*p* > 0.5), and the five-year OS was not statistically significantly different between the two treatment regimens. In the study by Fanfani [18], the three-year DFS and OS were 62.9% and 68.3% versus 63.2% and 67.7% for two treatment regimens (*p* > 0.5). The recurrence and death rates were 40.7% and 28.7% for chemoradiotherapy and 37.6% and 30.1% for surgery after chemoradiotherapy (*p* < 0.05) among patients with stage IIIA and IIIB disease. Surgery after chemoradiotherapy significantly reduced the recurrence and death rates without any effect on DFS or OS. Based on the report by Darus [19], the mean OS was 113.8 months (94.4–133.3 months) in the surgery group and 113.7 months (92.2–135.1 months) in the chemoradiotherapy group for patients with stage IB2 disease (*p* > 0.5). The mean disease-free interval was 113.8 months (89.8–137.8 months) and 113.2 months (93.9–132.5 months) for the two groups (*p* > 0.5). Surgery or brachytherapy could be performed depending on tumour shrinkage after external beam radiotherapy.

These studies indicate that the recurrence and death rates could be decreased by surgery after chemoradiotherapy/radiotherapy without affecting OS.

In this meta-analysis, the OS for radiotherapy/chemoradiotherapy combined with surgery was not increased compared with that for radiotherapy/chemoradiotherapy alone in patients with locally advanced cervical cancer. Hence, surgery is not recommended after radiotherapy/chemoradiotherapy. After excluding Daru’s data because the patients received surgery without brachytherapy, the final results for OS remained the same. The articles included in this meta-analysis mainly focused on stage IB-IIB disease, especially the report by Perez [14]; hence, we conducted a subgroup analysis and found that the OS of patients with stage IB-IIB disease was not improved by radiotherapy/chemoradiotherapy combined with surgery. In 2016, a multicenter cohort study on chemoradiotherapy combined with image-guided brachytherapy in locally advanced cervical cancer showed promising results. The three- and five-year actuarial LC, pelvic control (PC), cancer-specific survival (CSS), and OS were 91% and 89%, 87% and 84%, 79% and 73%, and 74% and 65%, respectively. The three- and five-year actuarial LC rates for patients with stage IB, IIB, and IIIB disease were 98% and 98%, 93% and 91%, and 79% and 75%, respectively. The three- and five-year actuarial PC for patients with stage IB, IIB, and IIIB disease was 96% and 96%, 89% and 87%, and 73% and 67%, respectively. The five-year actuarial rates of grade three-five morbidity were 5%, 7%, and 5% for the bladder, gastrointestinal tract, and vagina, respectively [20]. There were some analyses of patients who received surgery after chemoradiotherapy/radiotherapy without comparison to those who received radiotherapy/chemoradiotherapy alone. The nine-year DFS and OS rates were 81% and 85% for patients with stage IB2 to IVA disease [21], the two-year LC was 91.7% for stage IIB to IIIA disease [22], the five-year OS and DFS rates were 84% and 76% for IB2 to IVB adenocarcinoma [23], the five-year DFS and OS rates were 83% and 90% for patients with stage IB2, IIA and IIB disease [24], and the two- and five-year DFS rates were 80.4% and 72.2% for patients with stage IB2, IIA and IIB disease [25]. Chemoradiotherapy was associated with excellent survival outcomes without severe side effects for locally advanced cervical cancer.

Side effects were reported in six studies in this meta-analysis, with data unavailable in the studies by Leguevaque [12] and Morice [15]. Grade one-two and/or three-four side effects were available in some articles. The digestive and/or urinary systems were involved in some studies. Unfortunately, more details regarding grade and affected system were not available. Consequently, side effects were not evaluated due to uncertainties in the data extraction. In the articles included in this meta-analysis, the results regarding side effects were different. In the research by Keys, grade three and four side effects occurred at a rate of 10% in both groups. The frequency of side effects was higher in the surgery group (63% versus 56%) [13]. According to Darus, surgery did not increase toxicity, which was mainly grade one-two. More gastrointestinal toxicities occurred in the chemoradiotherapy group (41% versus 21%), but this difference was not statistically significant [19]. In the publication by Fanfani, urinary and gastrointestinal complications were more prevalent after radiotherapy than after surgery (14.3% versus 0%). Grade one-two side effects occurred in 57.1% and 8.2% of the cases in the radiotherapy and surgery groups, respectively (*p <* 0.05). The rate of vascular complications was 16.4% after surgery compared with 0% after radiotherapy. Grade one-two and three-four side effects occurred in 15% and 1.4% of the surgery group. Late vascular complications were similar in the two groups. Grade one-two and three-four urinary side effects occurred in 7.8% and 7.6% of the radiotherapy group and 9.6% and 4.1% of the surgery group. The rates of grade one-two and three-four gastrointestinal side effects were 6.5% and 2.6% in the radiotherapy group and 6.8% and 0% in the surgery group [18]. In the study by Perez, grade three side effects occurred in 5–11% and 8–12% of patients in the radiotherapy and surgery groups. In the radiotherapy group, the following rates of side effects were reported: rectovaginal fistula, 1.5%; proctitis, 1.1%; small intestine obstruction, 1.8%; urethral stricture, 1.8%; and vesicovaginal fistula, 0.9%. In the surgery group, small intestine obstruction/perforation occurred in 4.2% of the cases, urethral stricture in 2.6%, vesicovaginal fistula in 1.6%, and rectovaginal fistula in 1.3% [14]. In the research by Mazeron, the cumulative incidence of severe late morbidity was higher in the surgery group at two years (16.2% versus 4.3%) and at five years (2.5% versus 6.5%) [17]. In the study by Centina, grade one-two side effects in the surgery group included proctitis and cystitis (50%), and the rate of grade three-four side effects was 2%. Six patients had an infection 30 days after surgery. In total, 3.4% of the patients underwent unilateral lymphocyst resection or drainage. Overall, 2.3% of the patients had a uretero-cutaneous fistula treated with surgery and double J-stent positioning [16].

Other adverse effects of surgery were as follows. Hospital stay was prolonged by five days (range, four to six days). Median surgical time was four hours (four to six hours). Median blood loss was 450 ml (150–600 ml). Furthermore, 13.9% of patients received transfusions, 3.4% had a vascular laceration, 1.5% had a urethral tear, 2.3% had urethral stricture, 1.5% had wound dehiscence, and 1.5% had infection of the surgical wound [16]. According to the research by Fanfani, the median operation time was 240 min (90–400 min), blood loss was 275 ml (100–3000 ml), and the total operative hospital stay was eight days (four to 18 days) [18]. In developing countries, surgery increases the economic burden. Since the data were limited, more details remain to be investigated.

### Limitations

There are some limitations to this meta-analysis. First, different chemotherapy regimens were administered, such as cisplatin, capecitabin, 5FU, carboplatin and gemcitabine. The effects of different chemotherapy regimens are unclear, but chemotherapy plays a crucial role in the tumour response to radiotherapy. Second, tumour response after chemoradiotherapy is a key factor in the decision to perform surgery [26]. For example, in the study by Leguevaque, surgery was performed in only patients who had a CR or a residual tumour less than 50% of the initial tumour size after chemoradiotherapy. In the study by Morice, surgery was conducted in patients with a CR after chemoradiotherapy. Residual disease in the cervix at the end of radiotherapy is one factor to consider [27, 28] in decision-making regarding surgery after radiotherapy. The response to radiotherapy/chemoradiotherapy was evaluated and sometimes repeated by gynaecological examination and/or MRI. Third, it has been reported that positive nodes are an independent prognostic factor for OS [29]. A high rate of extra-cervical disease and pelvic and/or para-aortic node involvement is associated with more radiotherapy resistance and decreased OS despite a CR after chemoradiotherapy [28]. The need for detecting positive nodes is emphasized before surgery. PET-CT was more promising than MRI at detecting positive nodes [30], but repeat MRI can provide proof of residual disease [31]. Fourth, squamous cell carcinoma and adenocarcinoma were investigated in this meta-analysis without separating the results by pathology category. Fifth, data on DFS, PFS, LC and recurrence rate were not available for the meta-analysis, and these results are still unknown. Finally, although surgery remains the first option for local relapse or an incomplete response to radiotherapy, the indications for surgery after radiotherapy/chemoradiotherapy are still controversial.

### Prospects

In conclusion, surgery after radiotherapy/chemoradiotherapy is not recommended as it has no effect on the OS of patients with locally advanced cervical cancer, including those with stage IB2, IIA2, IIB, IIIA, IIIB or IVA disease. Further analysis showed that surgery is not recommended for patients with stage IB2, IIA2 or IIB disease. Side effects of surgery limit its application for locally advanced cervical cancer. More investigations on DFS, PFS, LC, recurrence rate and surgery indications are needed, especially for patients with stage IB2, IIA2 and IIB disease.

## Conclusions

This meta-analysis suggested that surgery had no effect on overall survival after radiotherapy/chemoradiotherapy. Side effects according to radiotherapy combined with surgery are not superior than radiotherapy/chemoradiotherapy. Surgery is not recommended for patients with locally advanced cervical cancer. Concurrent chemoradiotherapy is preferred for locally advanced cervical cancer.

## References

[1] Parkhurst JO, Vulimiri M (2013). Cervical cancer and the global health agenda: insights from multiple policy-analysis frameworks. Glob Public Health.

[2] Torre LA, Bray F, Siegel RL, Ferlay J, Lortet-Tieulent J, Jemal A (2015). Global cancer statistics, 2012. CA Cancer J Clin.

[3] Green JA, Kirwan JM, Tierney JF, Symonds P, Fresco L, Collingwood M, Williams CJ (2001). Survival and recurrence after concomitant chemotherapy and radiotherapy for cancer of the uterine cervix: a systematic review and meta-analysis. Lancet.

[4] Network NCC. Cervical Cancer [https://www.nccn.org/professionals/physician_gls/pdf/cervical.pdf]. Accessed 9 Aug 2017.

[5] Moher D, Liberati A, Tetzlaff J, Altman DG, Group P (2010). Preferred reporting items for systematic reviews and meta-analyses: the PRISMA statement. Int J Surg.

[6] Parmar MK, Torri V, Stewart L (1998). Extracting summary statistics to perform meta-analyses of the published literature for survival endpoints. Stat Med.

[7] Tierney JF, Stewart LA, Ghersi D, Burdett S, Sydes MR. Practical methods for incorporating summary time-to-event data into meta-analysis. Trials. 2007;(8):16. 10.1186/1745-6215-8-16 PMC1920534 17555582

[8] Williamson PR, Smith CT, Hutton JL, Marson AG (2002). Aggregate data meta-analysis with time-to-event outcomes. Stat Med.

[9] Higgins JP, Thompson SG (2002). Quantifying heterogeneity in a meta-analysis. Stat Med.

[10] Stuck AE, Rubenstein LZ, Wieland D. Bias in meta-analysis detected by a simple, graphical test. Asymmetry detected in funnel plot was probably due to true heterogeneity. BMJ. 1998;316(7129):469. author reply 470-61 PMC2665578 9492685

[11] GA Wells BS, D O'Connell, J Peterson, V Welch, M Losos, P Tugwell. The Newcastle-Ottawa Scale (NOS) for assessing the quality of nonrandomised studies in meta-analyses. Appl Eng Agric. 2012;18(6):727–34.

[12] Leguevaque P, Motton S, Delannes M, Querleu D, Soule-Tholy M, Tap G, Houvenaeghel G (2011). Completion surgery or not after concurrent chemoradiotherapy for locally advanced cervical cancer?. Eur J Obstet Gynecol Reprod Biol.

[13] Keys HM, Bundy BN, Stehman FB, Okagaki T, Gallup DG, Burnett AF, Rotman MZ, Fowler WC (2003). Radiation therapy with and without extrafascial hysterectomy for bulky stage IB cervical carcinoma: a randomized trial of the gynecologic oncology group. Gynecol Oncol.

[14] Perez CA, Grigsby PW, Camel HM, Galakatos AE, Mutch D, Lockett MA (1995). Irradiation alone or combined with surgery in stage IB, IIA, and IIB carcinoma of uterine cervix: update of a nonrandomized comparison. Int J Radiat Oncol Biol Phys.

[15] Morice P, Rouanet P, Rey A, Romestaing P, Houvenaeghel G, Boulanger JC, Leveque J, Cowen D, Mathevet P, Malhaire JP (2012). Results of the GYNECO 02 study, an FNCLCC phase III trial comparing hysterectomy with no hysterectomy in patients with a (clinical and radiological) complete response after chemoradiation therapy for stage IB2 or II cervical cancer. Oncologist.

[16] Cetina L, Gonzalez-Enciso A, Cantu D, Coronel J, Perez-Montiel D, Hinojosa J, Serrano A, Rivera L, Poitevin A, Mota A (2013). Brachytherapy versus radical hysterectomy after external beam chemoradiation with gemcitabine plus cisplatin: a randomized, phase III study in IB2-IIB cervical cancer patients. Ann Oncol.

[17] Mazeron R, Gouy S, Chargari C, Rivin Del Campo E, Dumas I, Mervoyer A, Genestie C, Bentivegna E, Balleyguier C, Pautier P (2016). Post radiation hysterectomy in locally advanced cervical cancer: outcomes and dosimetric impact. Radiother Oncol.

[18] Fanfani F, Vizza E, Landoni F, de Iaco P, Ferrandina G, Corrado G, Gallotta V, Gambacorta MA, Fagotti A, Monterossi G (2016). Radical hysterectomy after chemoradiation in FIGO stage III cervical cancer patients versus chemoradiation and brachytherapy: complications and 3-years survival. Eur J Surg Oncol.

[19] Darus CJ, Callahan MB, Nguyen QN, Pastore LM, Schneider BF, Rice LW, Jazaeri AA (2008). Chemoradiation with and without adjuvant extrafascial hysterectomy for IB2 cervical carcinoma. Int J Gynecol Cancer.

[20] Sturdza A, Potter R, Fokdal LU, Haie-Meder C, Tan LT, Mazeron R, Petric P, Segedin B, Jurgenliemk-Schulz IM, Nomden C (2016). Image guided brachytherapy in locally advanced cervical cancer: improved pelvic control and survival in RetroEMBRACE, a multicenter cohort study. Radiother Oncol.

[21] Jurado M, Martinez-Monge R, Garcia-Foncillas J, Azinovic I, Aristu J, Lopez-Garcia G, Brugarolas A (1999). Pilot study of concurrent cisplatin, 5-fluorouracil, and external beam radiotherapy prior to radical surgery +/− intraoperative electron beam radiotherapy in locally advanced cervical cancer. Gynecol Oncol.

[22] Mancuso S, Smaniotto D, Benedetti Panici P, Favale B, Greggi S, Manfredi R, Margariti PA, Morganti AG, Scambia G, Tortoreto F (2000). Phase I-II trial of preoperative chemoradiation in locally advanced cervical carcinoma. Gynecol Oncol.

[23] Shibata K, Kajiyama H, Yamamoto E, Terauchi M, Ino K, Nomura S, Nawa A, Kawai M, Kikkawa F (2009). Effectiveness of preoperative concurrent chemoradiation therapy (CCRT) for locally advanced adenocarcinoma of cervix. Eur J Surg Oncol.

[24] Ferrandina G, Legge F, Fagotti A, Fanfani F, Distefano M, Morganti A, Cellini N, Scambia G (2007). Preoperative concomitant chemoradiotherapy in locally advanced cervical cancer: safety, outcome, and prognostic measures. Gynecol Oncol.

[25] Huguet F, Cojocariu OM, Levy P, Lefranc JP, Darai E, Jannet D, Ansquer Y, Lhuillier PE, Benifla JL, Seince N (2008). Preoperative concurrent radiation therapy and chemotherapy for bulky stage IB2, IIA, and IIB carcinoma of the uterine cervix with proximal parametrial invasion. Int J Radiat Oncol Biol Phys.

[26] Morice P, Uzan C, Zafrani Y, Delpech Y, Gouy S, Haie-Meder C (2007). The role of surgery after chemoradiation therapy and brachytherapy for stage IB2/II cervical cancer. Gynecol Oncol.

[27] Classe JM, Rauch P, Rodier JF, Morice P, Stoeckle E, Lasry S, Houvenaeghel G (2006). Surgery after concurrent chemoradiotherapy and brachytherapy for the treatment of advanced cervical cancer: morbidity and outcome: results of a multicenter study of the GCCLCC (Groupe des Chirurgiens de Centre de Lutte Contre le Cancer). Gynecol Oncol.

[28] Azria E, Morice P, Haie-Meder C, Thoury A, Pautier P, Lhomme C, Duvillard P, Castaigne D (2005). Results of hysterectomy in patients with bulky residual disease at the end of chemoradiotherapy for stage IB2/II cervical carcinoma. Ann Surg Oncol.

[29] Touboul C, Uzan C, Mauguen A, Gouy S, Rey A, Pautier P, Lhomme C, Duvillard P, Haie-Meder C, Morice P (2010). Prognostic factors and morbidities after completion surgery in patients undergoing initial chemoradiation therapy for locally advanced cervical cancer. Oncologist.

[30] Uzan C, Vincens E, Balleyguier C, Gouy S, Pautier P, Duvillard P, Haie-Meder C, Morice P (2010). Outcome of patients with incomplete resection after surgery for stage IB2/II cervical carcinoma with chemoradiation therapy. Int J Gynecol Cancer.

[31] Vincens E, Balleyguier C, Rey A, Uzan C, Zareski E, Gouy S, Pautier P, Duvillard P, Haie-Meder C, Morice P (2008). Accuracy of magnetic resonance imaging in predicting residual disease in patients treated for stage IB2/II cervical carcinoma with chemoradiation therapy : correlation of radiologic findings with surgicopathologic results. Cancer.

